# Guard Cell Microfilament Analyzer Facilitates the Analysis of the Organization and Dynamics of Actin Filaments in *Arabidopsis* Guard Cells

**DOI:** 10.3390/ijms20112753

**Published:** 2019-06-05

**Authors:** Xin Li, Min Diao, Yanan Zhang, Guanlin Chen, Shanjin Huang, Naizhi Chen

**Affiliations:** 1 Key Laboratory of Plant Resources, Institute of Botany, Chinese Academy of Sciences, Beijing 100093, China; lixinqiu@126.com; 2 University of Chinese Academy of Sciences, Beijing 100049, China; 3 Center for Plant Biology, School of Life Sciences, Tsinghua University, Beijing 100084, China; diaomin@shanghaitech.edu.cn; 4 iHuman Institute, Shanghai Tech University, Shanghai 201210, China; 5 OLYMPUS (CHINA) CO., LTD, Beijing 100027, China; nanknight@foxmail.com; 6 Baidu Online Network Technology (Beijing) CO., LTD, Beijing 100193, China; hackinfer@gmail.com

**Keywords:** guard cell microfilament angle (GCMA), individual actin filaments, different orientations, actin dynamics, technical advance, *Arabidopsis*

## Abstract

The actin cytoskeleton is involved in regulating stomatal movement, which forms distinct actin arrays within guard cells of stomata with different apertures. How those actin arrays are formed and maintained remains largely unexplored. Elucidation of the dynamic behavior of differently oriented actin filaments in guard cells will enhance our understanding in this regard. Here, we initially developed a program called ‘guard cell microfilament analyzer’ (GCMA) that enables the selection of individual actin filaments and analysis of their orientations semiautomatically in guard cells. We next traced the dynamics of individual actin filaments and performed careful quantification in open and closed stomata. We found that de novo nucleation of actin filaments occurs at both dorsal and ventral sides of guard cells from open and closed stomata. Interestingly, most of the nucleated actin filaments elongate radially and longitudinally in open and closed stomata, respectively. Strikingly, radial filaments tend to form bundles whereas longitudinal filaments tend to be removed by severing and depolymerization in open stomata. By contrast, longitudinal filaments tend to form bundles that are severed less frequently in closed stomata. These observations provide insights into the formation and maintenance of distinct actin arrays in guard cells in stomata of different apertures.

## 1. Introduction

The size of stomatal pores, each surrounded by a pair of guard cells, regulates CO_2_ uptake and water loss, which is controlled by stomatal movement [1,2]. Tight regulation of stomatal closure and opening is therefore crucial for plant growth and development as well as the interactions of plants with the surrounding environment. The actin cytoskeleton has been implicated in the regulation of stomatal closure and opening [3,4,5,6,7,8,9,10]. It was shown that actin is involved in the regulation of ion transportation, chloroplast position, and vacuole fusion in guard cells [11,12,13,14,15,16], which provide insights into the mechanism of action of actin during stomatal movement. However, how exactly actin regulates stomatal closure and opening remains largely unknown.

The cellular functions of the actin cytoskeleton are dictated by its spatial organization and dynamics in cells [17]. The organization of actin filaments (also known as microfilaments) in guard cells has been revealed with the approaches of fluorescent phalloidin-staining and immunostaining with anti-actin antibody in fixed guard cells at earlier days [3,18,19], and decoration with actin markers in living guard cells [4,5,6,7,8,9,16,20]. In order to better understand the organization and function of actin in guard cells, the colleagues in this field have tried to quantify the overall organization of actin filaments within guard cells. For instance, Hagaki et al. divided stomata into four different classes based on the patterns of the metrics of MF orientation and characterized their organization by several useful parameters, such as actin filament orientation or angular difference, bundling or skewness, and density or occupancy [5]. Given that guard cells have unique geometry and undergo changes during stomatal closure and opening, how to define the radial, longitudinal, and oblique orientation of individual actin filaments has not been solved clearly. Nonetheless, using this approach, Shimono et al. examined the behavior of actin filaments in guard cells in response to the activation of immune signaling and found that the purified pathogen elicitor flg22 (a highly conservative peptide of 22 amino acids in N terminus of bacteria flagellin) and chitin treatments induced some specific changes in actin configurations [9], but the underlying details of single filament dynamics remain unexplored. Nonetheless, these results revealed that actin filaments form distinct actin arrays at different stomatal apertures. However, how these distinct actin arrays are constructed and maintained remains to be documented.

Direct visualization of the dynamics of individual actin filaments within guard cells is going to yield insights into this question. Indeed, several previous studies revealed that it is possible to trace the dynamics of individual actin filaments within guard cells [8,14], but how the dynamic behavior of individual actin filaments contributes to the construction and maintenance of actin arrays in guard cells remains to be determined. Again, considering that guard cells have unique geometry and undergo changes during stomatal closure and opening, it is important to develop a program which enables automatic selection of actin filaments and is able to link to their positional information. Development of such a program will facilitate the analysis of how the dynamic behavior of individual actin filaments contributes to the generation and maintenance of distinct actin arrays in guard cells of stomata at different apertures.

In this study, we initially designed a program in MATLAB software to recognize the skeletonized actin filaments (lines) semiautomatically that allows the calculation of the value of angle of each filament (line) relative to the edge of stomatal pore. We categorized the stomata into four stages based on the ratio of width/length of the stomatal pore. We then performed careful characterization of the overall organization and dynamics of individual actin filaments within guard cells and found that actin filaments are arrayed into distinct structures within guard cells of stomata at different stages. Live cell imaging of the dynamics of individual actin filaments showed that longitudinally oriented actin filaments tend to be removed whereas radially oriented actin filaments tend to form actin bundles that are stabilized and retained in guard cells of open stomata. By contrast, longitudinally oriented actin filaments tend to form actin bundles that are stabilized and retained in guard cells of closed stomata. This report represents the first to separately analyze the dynamics of actin filaments in different orientations within guard cells of stomata at different apertures. Our study provides insights into the organization of actin filaments and maintenance of actin arrays within guard cells.

## 2. Results

### 2.1. Guard Cell Microfilament Analyzer (GCMA) Allows Semiautomatic Measurements of Angles of Individual Actin Filaments Formed with the Stomatal Pore Edge in the Guard Cell

The spatial distribution and dynamics of the actin cytoskeleton are crucial for its functions. To understand the role of actin in regulating stomatal closure and opening, we need to reveal the details underlying the organization and dynamics of the actin cytoskeleton in guard cells. In this regard, development of assays to analyze the organization of actin filaments in guard cells is critical.

**Figure 1:**
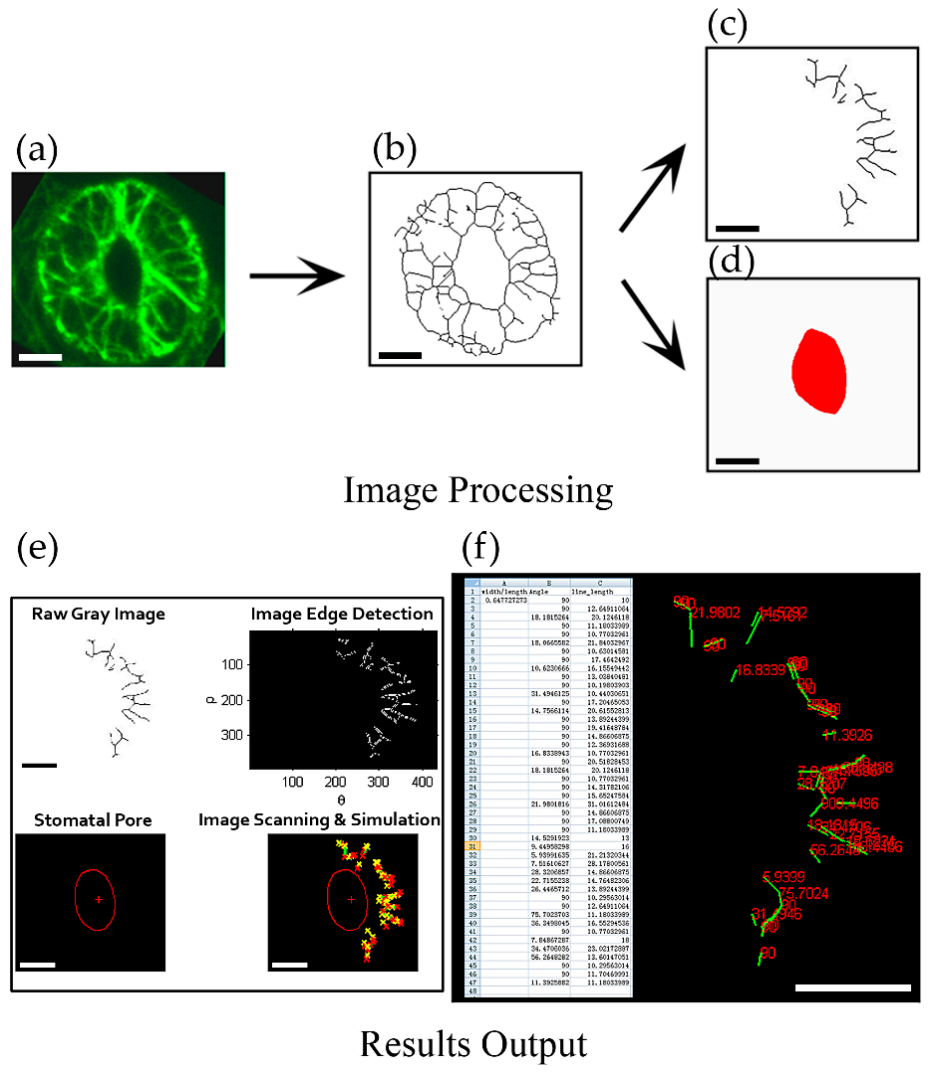
Framework of quantification of angle values in individual actin filaments in guard cell. (**a**) The obtained image of the maximum intensity projection of serial optical sections with GFP-marked actin filaments in guard cell; (**b**) The skeletonized image of actin filaments in (**a**) [5]. (**c**) Dividing the skeletonized filaments of the guard cell in the right part of stomata and saving it as a JPEG format file. (**d**) Drawing the stomatal pore region by red color and saving it as a JPEG format file. (**e**) Image detection and identification after inputting two images of the skeletonized filaments (**c**) and the stomatal pore region (**d**) into GCMA software (see details in supplemental PPT). The image on the upper left was the grayscale image of the skeletonized filaments in (**c**) and the image on the upper right was the edge detection image of the skeletonized filaments. The image on the lower left showed the recognized stomatal pore in (**d**) and the image on the lower right showed the merge image of the simulated stomatal pore and skeletonized filaments. (**f**) Result output and data mining. Excel table on the left showed the metrics of angles and lengths of individual filaments, as well as the value of width/length of the stomata. The right part showed the values of angles (red numbers) corresponding to each of recognized filaments (green lines) in the output result. Bars = 5 μm in (a)—(d). Bars = 100 pixels in (e)—(f).

To achieve this goal, we designed a program named ‘guard cell microfilament analyzer ’(GCMA) using MATLAB code that can reveal the orientation of actin filaments relative to the stomatal pore edge. It can recognize every individual skeletonized filament (line) in one guard cell semiautomatically. Because in different positions outside stomatal pore, the radial directions to the stomatal pore edge are different (Supplemental Figure S2). The spatial information of actin filaments was revealed by determining the angles formed between actin filaments and their respective radial lines to the stomatal pore edge (Supplemental Figure S3). To preprocess the raw image data, we skeletonized the fluorescent image of actin filaments in guard cells firstly (Figure 1a,b, see the detailed method in [5]). Filamentous lines and the stomatal pore region were subsequently divided into two independent images (Figure 1c,d) that were input into the GCMA program. Once the image is recognized by the GCMA program, the simulated filaments and stomatal pore will be output (Figure 1e), so does the value of angle of each filament (Figure 1f). Finally, the values of angles, filament lengths, and stomatal aperture (width/length), will be completely summarized and output into an Excel table (Figure 1f) under the same folder with the file of GCMA.exe. During the usage process, the parameters can be adjusted to make the filament lines and the stomatal pore easily recognizable (Supplemental Figure S1b,e). The detailed procedures of the installation of the program and how to change parameters to recognize the filament lines and the red stomatal pore can be found in Supplemental PPT and Supplemental Figure S1.

Next, to test the validity of the GCMA program, we measured the values of angles of hand-draw actin filaments in open stomata and closed stomata respectively by GCMA program. It can be seen from the output results that one filament showed the angle value of 22 degrees in open stomata, whereas the filament in the corresponding position showed the angle value of 57 degrees in closed stomata (see filaments indicated by yellow arrows in Supplemental Figure S2a,b,g,h). The outputting values of angles for another filament (indicated by yellow arrows in Supplemental Figure S2c,d,i,j) was 90 degrees and 47 degrees in open stomata and closed stomata, respectively. These results confirmed that this GCMA program can measure the angles of individual filaments relative to the stomatal pore edge. Therefore, the GCMA program is a very powerful tool for measuring the values of angles of actin filaments to indicate their spatial information in term of their orientations relative to the stomatal pore edge in a guard cell. It can avoid subjective bias and provide more objective data through identifying the filaments semiautomatically. Classification of the orientations of actin filaments using GCMA program will facilitate the study of the organization and dynamics of actin filaments in guard cells.

### 2.2. The Amount of Longitudinally Oriented Actin Filaments in Guard Cells Gradually Increases with the Closing of Stomata during Diurnal Cycle

**Figure 2:**
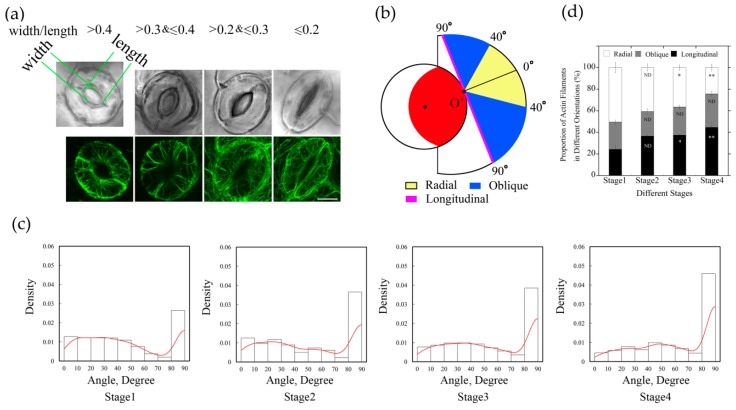
The proportion of longitudinally oriented actin filaments in guard cells increased during diurnal stomatal closure. (**a**) Typical actin arrays in guard cell at four stages of diurnal stomata. According to the ratio of width/length, stomata were classified into four stages. Stage 1, width/length > 0.4, Stage 2, 0.3 < width/length ≤ 0.4, Stage 3, 0.2 < width/length ≤ 0.3, Stage 4, width/length ≤ 0.2. Bar = 5 μm. (**b**) Schematic diagram describing classification of actin filaments based on the values of actin filament angles. Actin filaments were grouped into three classes, radial (0° ≤ angle < 40°), oblique (40° ≤ angle < 90°), and longitudinal (angle = 90°) orientation. The red region indicates the stomatal pore, the inner black circle represents the virtual circle through either the edge of the ventral wall of the guard cell or the edge of stomatal pore. For example, if the actin filaments or the extension lines of the actin filaments intersected with stomatal pore edge at point O’, actin filaments in yellow regions and blue regions indicate that they are radial and oblique relative to the stomatal pore, respectively. Otherwise, if the actin filaments or their extension lines are tangential to the stomatal pore edge, such as the magenta line, or do not intersect with the stomatal pore edge, they belong to the longitudinal orientation. (**c**) The angles formed between actin cables and their respective radial directions of stomatal pore increased during diurnal stomatal closure. The histograms of the distribution of angles from Stage 1 to Stage 4 stomata are presented from left to right. Red lines are the kernel density estimation of the angle values distribution. At least 600 actin cables from 40 guard cells were selected for the measurement. (**d**) Radially oriented actin filaments in guard cell accounted for the most in open stomata, whereas longitudinally oriented actin filaments accounted for the most in closed stomata. According to the values of angles of individual actin filaments at four stages of stomata, the proportions of differently oriented actin filaments (radial, oblique, and longitudinal) were analyzed based on the classification described in (**b**). ND, no significant difference; * *P* < 0.05; ** *P* < 0.01, which were compared with actin filaments in the same orientation at Stage 1 of open stomata by chi-square test. The results were the average in three repeats ± SE.

In order to further understand the arrangement of actin filaments relative to stomatal apertures, we divided stomata into four stages during diurnal cycle based on the values of stomatal apertures. Among them, the Stage 1 stomata with the ratio of width/length greater than 0.4; the Stage 2 stomata with the ratio of width/length between 0.3–0.4; the Stage 3 stomata with the ratio of width/length between 0.2–0.3; and the Stage 4 stomata with the ratio of width/length was equal or less than 0.2 (Figure 2a). Then we measured the angles of each filament using GCMA program. The histogram distribution of the values of angles of actin filaments within guard cell showed that values of angles of actin filaments are mainly in the range of 0–40 degrees, and the actin array mainly showed a radial arrangement in Stage 1 stomata (Figure 2a,c). With the decrease in stomatal aperture, the proportion of single filaments with the value of angle at 90 degrees increased, and the peak value of angles gradually shifted from 0–40 degrees to 30–40 degrees at Stage 3, which indicated that the proportion of actin filaments in radial distribution decreased. At Stage 4, the proportion of 90-degree filaments further increased, and the peak value of angles of actin filaments was at 40–50 degrees, indicating that the proportion of longitudinal filaments increased with the decrease in stomatal aperture (Figure 2c). These results indicated that the amount of longitudinally oriented actin filaments increases during the closing of stomata.

We found that the proportion of actin filaments with angles around 40 degrees changed more obviously from Stage 1 to Stage 4 (Figure 2c), therefore, we defined 40 degrees as the boundary value that distinguished radial actin filaments from oblique actin filaments. When the skeletonized filament or the extending line of skeletonized filament did not intersect with the stomatal pore edge, the value of the angle was defined as 90 degrees and classified as longitudinal orientation. Based on the values of angles of those actin filaments, they can be grouped into three classes: radial (R) orientation, oblique (O) orientation, and longitudinal (L) orientation (Figure 2b).

According to the measured values of angles formed between actin filaments and their respective radial lines related to the stomatal pore edge (Supplemental Figure S3), actin filaments in guard cells at each stage were analyzed and classified by their orientations. From the statistical analysis of angle values in different stages of stomatal closure measured by GCMA program, it can be seen easily that the proportion of individual actin filaments in radial orientation was greatest in open stomata, and decreased in closed stomata, whereas the longitudinal filaments increased gradually in diurnal stomatal closure from Stage 1 stomata to Stage 4 stomata (Figure 2d). With the change in stomatal apertures, the proportion of actin filaments at three different orientations changed as well. It can be concluded that the composition of actin filaments with different orientations accounts for the formation of different actin arrays in guard cells of stomata at different stages.

### 2.3. Actin Nucleation Occurs at both Dorsal and Ventral Sides of Guard Cells, and the Majority of Actin Filaments Elongate Radially and Longitudinally in Guard Cells of Open and Closed Stomata, Respectively

**Figure 3:**
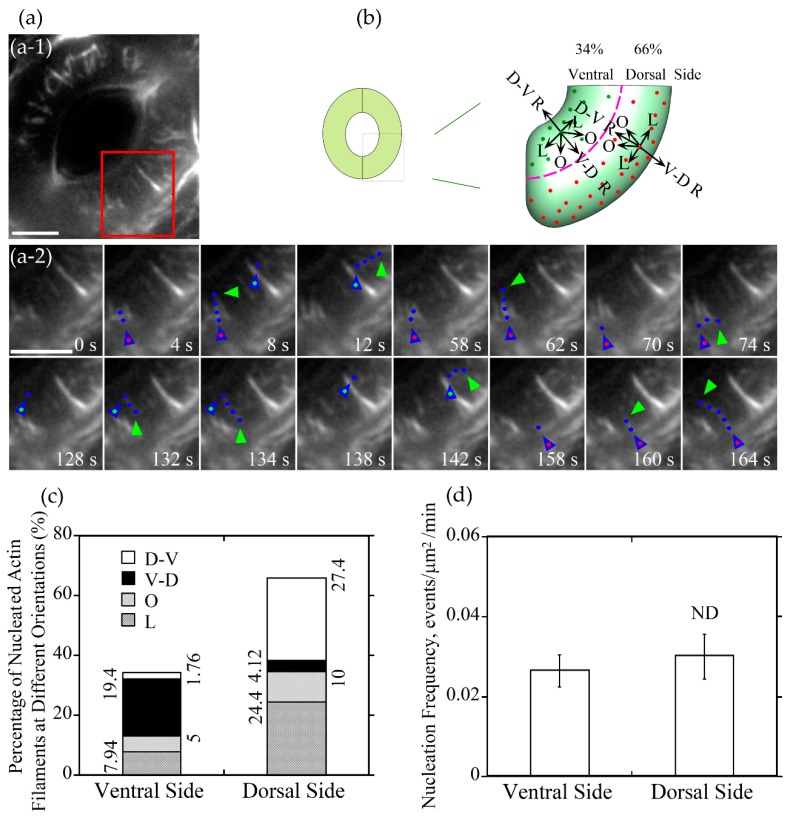
Actin nucleation occurs at both dorsal and ventral sides and actin filaments elongate mainly in radial orientation in guard cells of open stomata. (**a**) Dynamics of actin nucleation and elongation of single actin filaments in guard cell of open stomata in wild-type (WT). (**a**-**1**) Overview of the open stomata expressing GFP-ABD2-GFP, the red box marked the region of interest. (**a**-**2**) An enlarged view of time-lapse images of actin nucleation and elongating direction in the region of interest in (**a**-**1**). Blue triangles with red dot inside indicate the nucleation sites or the starting points of the elongating actin filaments in the dorsal side of guard cells, and blue triangles with green dot inside indicate the nucleation sites or the starting points of the elongating actin filaments in the ventral side of guard cells. Blue dots indicate the elongating actin filaments. Green triangles indicate the newly elongated direction of actin filaments. Bar = 5 μm. (**b**) Schematic diagram describing actin filaments of four different orientations (D-V R, dorsal-to-ventral radial; V-D R, ventral-to-dorsal radial; O, oblique; L, longitudinal) in dorsal and ventral side of guard cells in open stomata. The dotted pink line is the boundary between dorsal and ventral region of the guard cell. Red and green dots represent actin nucleation sites in dorsal and ventral region of the guard cell, respectively. Arrows indicate the direction of the elongation of the actin filaments. (**c**) The proportion of actin filaments at each orientation (D-V R, dorsal-to-ventral radial; V-D R, ventral-to-dorsal radial; O, oblique; L, longitudinal) in the dorsal side and ventral side of guard cells in open stomata. (**d**) Quantification of actin nucleation frequency. Actin nucleation frequency was defined as number of actin nucleation events per unit area per unit time (events/μm^2^/min). ND, no significant difference. See Supplemental Movie 1 online for the entire series in (**a**). Bar = 5 μm.

**Figure 4:**
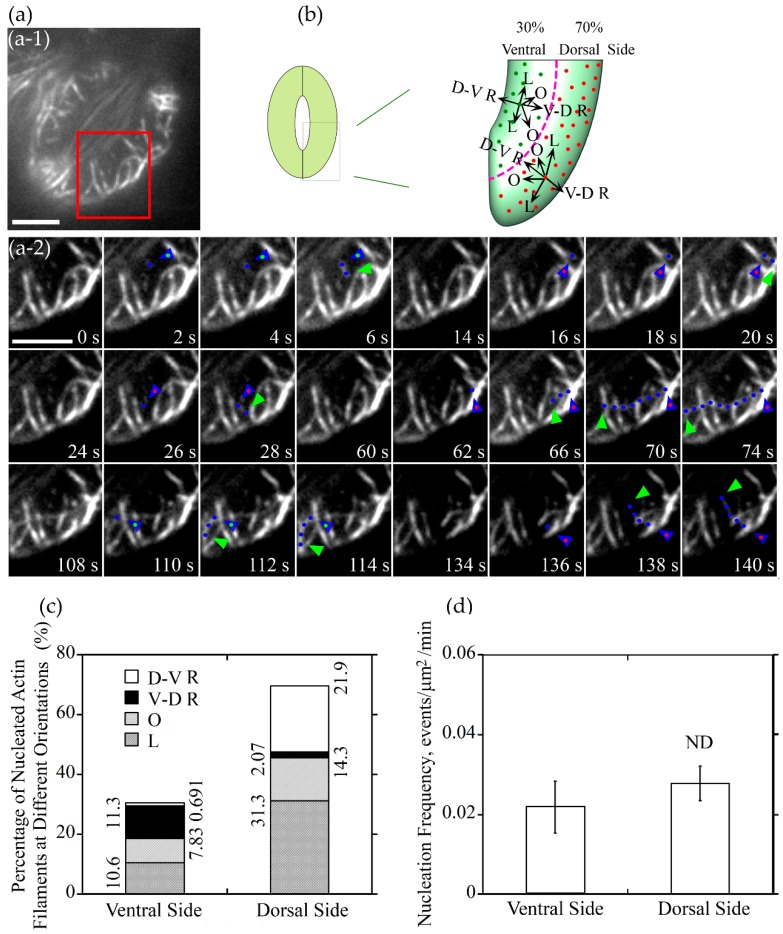
Actin nucleation occurs at both of dorsal side and ventral side of guard cells, and the majority of them elongated along the longitudinal orientation in closed stomata. (**a**) Dynamics of actin nucleation and elongation of single actin filaments in guard cell of closed stomata in wild type (WT). (**a**-**1**) Overview of the closed stomata expressing GFP-ABD2-GFP, the red box marked the region of interest. (**a**-**2**) An enlarged view of time-lapse images of actin nucleation and elongating direction in the region of interest in (**a**-**1**). Blue triangles with red dot inside indicate the nucleation sites or the starting points of the elongating actin filaments in the dorsal side of guard cells, and blue triangles with green dot inside indicate the nucleation sites or the starting points of the elongating actin filaments in the ventral side of guard cells. Blue dots indicate the elongating actin filaments. Green triangles indicate the newly elongated direction of actin filaments. Bar = 5 μm. (**b**) Schematic diagram describing actin filaments of different orientations (D-V R, dorsal-to-ventral radial; V-D R, ventral-to-dorsal radial; O, oblique; L, longitudinal) in the dorsal side and ventral side of guard cells in closed stomata. The dotted pink line is the boundary between dorsal region and ventral region of the guard cell. Red and green dots represent actin nucleation sites in dorsal and ventral region of the guard cell, respectively. Arrows indicate the direction of the elongation of the actin filaments. (**c**) The proportion of actin filaments of each orientation (D-V R, dorsal-to-ventral radial; V-D R, ventral-to-dorsal radial; O, oblique; L, longitudinal) in the dorsal side and ventral side of guard cells in closed stomata. (**d**) Nucleation frequency per unit area showed no significant difference between dorsal side and ventral side of guard cell in closed stomata. ND, no significant difference. See Supplemental Movie 2 online for the entire series in (**a**). Bar = 5 μm.

To understand how different actin arrays are generated and maintained within guard cells of stomata at different stages, we performed live-cell imaging of actin dynamics in guard cells. We initially traced where actin nucleation occurs in guard cells. We divided the guard cell into two parts by a longitudinally central line of the guard cell (marked with a dotted pink line in Figure 3b and Figure 4b), one is near the dorsal side (red dots marked nucleation sites in Figure 3b and Figure 4b) and the other is near the ventral side (green dots marked nucleation sites in Figure 3b and Figure 4b). We found that the proportion of actin nucleation sites in the dorsal and ventral region of guard cells of open stomata was 66% and 34%, respectively (Figure 3a,b,c), whereas the proportion of actin nucleation sites in the dorsal and ventral region of guard cells of closed stomata was 70% and 30%, respectively (Figure 4a,b,c). Although the number of nucleation sites in dorsal region was much more than that in the ventral region in both open (Figure 3a,b,c) and closed (Figure 4a,b,c) stomata, there is no significant difference in the actin nucleation frequency between dorsal region and ventral region in open and closed stomata (Figure 3d and Figure 4d).

Next, we divided the newly nucleated actin filaments into four classes based on the orientations of their elongation: dorsal-to-ventral radial (D-V R) actin filament which elongates along the orientation from the dorsal side to the ventral side of the guard cell radially, ventral-to-dorsal radial (V-D R) actin filament which elongates along the orientation from the ventral side to the dorsal side of the guard cell radially, longitudinal (L) actin filament and oblique (R) actin filament in the dorsal and ventral region (Figure 3b and Figure 4b). The proportion of dorsal-to-ventral radially oriented actin filaments is 27.4% that accounted for the most of actin filaments nucleated at the dorsal side of guard cells in open stomata, and the proportion of the ventral-to-dorsal radially oriented actin filaments nucleated at ventral side is 19.4% that accounted for the most at the ventral side in open stomata (Figure 3c). Totally, the proportion of radial actin filaments is 46.8% in guard cells of open stomata. We found that the longitudinally oriented actin filaments and obliquely oriented actin filaments occupied about 32.34% and 15.0% of total actin filaments in guard cells of open stomata (Figure 3c). In summary, our study showed that radially elongated actin filaments occupied most of the newly nucleated actin filaments in guard cells of open stomata, which to some extent explains why actin filaments form radial array in guard cells of open stomata.

We also analyzed the orientation of the newly nucleated actin filaments in guard cells of closed stomata, actin filaments can be categorized into four classes in guard cells (see the schematic graph shown in Figure 4b). Longitudinally oriented actin filaments occupied 31.3% at the dorsal side of guard cells in closed stomata (Figure 4c). Considering along with the data that about 10.6% actin filaments elongate longitudinally at the ventral side, the total longitudinally oriented actin filaments occupied 41.9% in guard cells of closed stomata (Figure 4c). In addition, the obliquely oriented actin filaments occupied 22.13% of the total actin filaments, including 14.3% and 7.83% of actin filaments at dorsal and ventral side, respectively (Figure 4c). These data suggest that longitudinally oriented actin filaments occupied most of the newly nucleated actin filaments in guard cells of closed stomata, which to some extent explains why actin filaments are arrayed into longitudinal arrangement in guard cells of closed stomata.

### 2.4. Longitudinally Oriented Actin Filaments Elongate Substantially Faster than Actin Filaments in Other Orientations in Guard Cells of Closed Stomata

**Figure 5:**
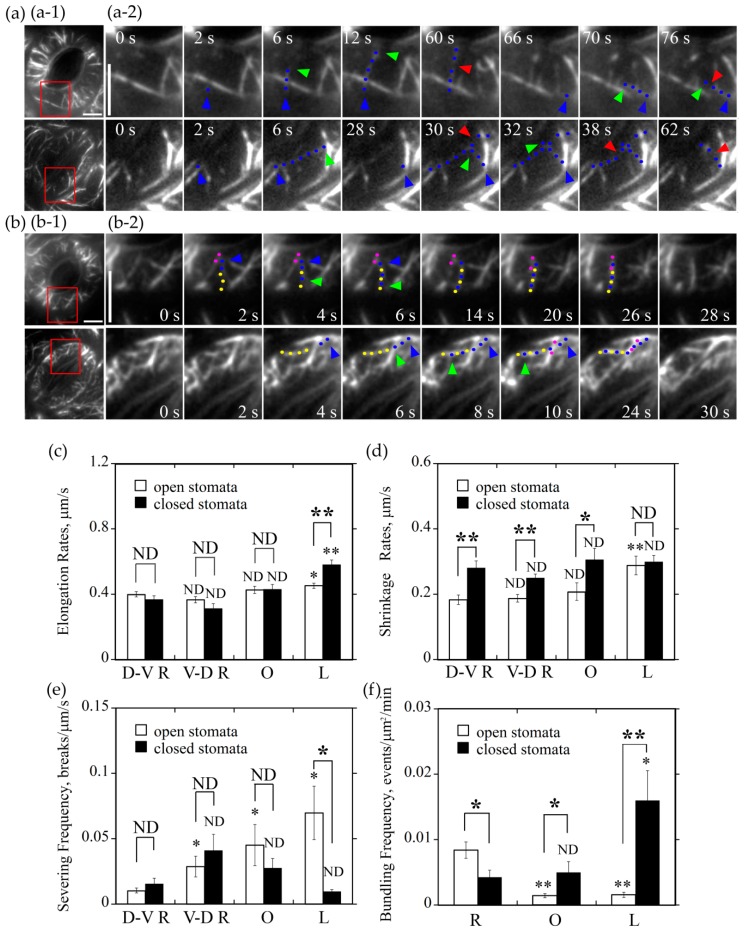
Radially oriented actin filaments tend to form bundles in guard cells of open stomata whereas longitudinally oriented actin filaments tend to form actin bundles in guard cells of closed stomata. (**a**) Longitudinally oriented actin filaments were prone to be severed in guard cells of open stomata but not in closed stomata. (**a**-**1**) Overviews of the organization of actin filaments decorated by GFP-ABD2-GFP in guard cells of open and closed stomata. The red box marked the region of interest. (**a**-**2**) Time-lapse series showed the severing activity of actin filaments in longitudinal orientation was higher than other oriented filaments in guard cells of open stomata but not in closed stomata. In open stomata, one actin filament that elongates from the starting point at 2 s to the ending point at 12 s, which indicated a dorsal-to-ventral radially oriented actin filament and it was severed at 60 s. Another actin filament elongates from the starting point at 66 s to the ending point at 70 s, which indicated the longitudinally oriented actin filament and it was severed at 76 s. However, in closed stomata, one actin filament that elongates from the starting point at 2 s to the ending point at 6 s, which indicated a longitudinally oriented actin filament and it was severed at 30 s and 38 s. Another actin filament elongates from the starting point at 28 s to the ending point at 32 s that indicated a dorsal-to-ventral radially oriented actin filament, which was severed at 62 s. Blue triangles represent the nucleation sites of actin filaments, blue dots represent the elongating actin filaments, and red triangles represent the severing points of the actin filaments, respectively. Bar = 5 μm. See Supplemental Movies 3 and 4 for the entire series. Time points indicate elapsed time from the start time of the movie sequence. Bar = 5 μm. (**b**) Formation of radially and longitudinally oriented actin bundles in open and closed stomata. (**b**-**1**) Overviews of actin filaments decorated with GFP-ABD2-GFP in guard cells of the open and closed stomata, and the red box marked the region of interest. (**b**-**2**) Time-lapse images showing the formation of radial actin bundles in open stomata and longitudinal actin bundles in closed stomata. Pink and yellow dot lines represent different preexisting actin cables. Blue dot lines represent the elongating actin filaments. Blue triangles represent the nucleation sites or the starting points of the elongation of actin filaments. Green triangles represent the elongating directions of the actin filaments. Bar = 5 μm. See Supplemental Movies 5 and 6 for the entire series. Time points indicate elapsed time from the beginning of the movie sequence. Bar = 5 μm. (**c**) Longitudinally oriented actin filaments elongated faster than actin filaments in other orientations in guard cells of closed stomata, but also faster than the longitudinally oriented actin filaments in guard cells of open stomata. (**d**) Longitudinally oriented actin filaments shrank faster than actin filaments in other orientation in guard cells of open stomata, but it is not the case in closed stomata. (**e**) Longitudinally oriented actin filaments were subject to severing with higher frequency than actin filaments in other orientations in guard cells of open stomata, but it is not the case in closed stomata. (**f**) The bundling frequency of radially oriented actin filaments was highest in guard cells of open stomata, whereas bundling frequency of longitudinally oriented actin filaments was the highest in guard cells of closed stomata. * *P* < 0.05 and ** *P* < 0.01; ND, no significant difference by a Student’s *t* test. D-V R actin filament (**c**,**d**,**e**) or R actin filament (**f**) served as the control except the specific comparisons marked by lines between the two columns in each graph.

We also measured the elongation rate of newly nucleated actin filaments that elongate at different orientations. We found that D-V R, V-D R, and O actin filaments elongate at approximately 0.40 μm/s in guard cells of open stomata. By comparison, actin filaments in L orientation elongated slightly faster with the average elongation rate reached at 0.45 μm/s in guard cells of open stomata (open hole columns in Figure 5c, *P* < 0.05). In guard cells of closed stomata, longitudinally oriented actin filaments elongate at the average elongation rate of 0.58 μm/s, which is much faster than that of actin filaments at other three orientations, whose elongation rate was approximately 0.4 μm/s (black hole columns in Figure 5c, *P* < 0.01). Interestingly, we found that there is no obvious difference in elongation rates of D-V R, V-D R, O actin filaments between open and closed stomata (Figure 5c, ND), except that L actin filaments elongated much faster in closed stomata than that in open stomata (Figure 5c, *P* < 0.01). Our data suggest that longitudinally oriented actin filaments elongate faster than actin filaments in other orientations.

### 2.5. Longitudinally Oriented Actin Filaments Tend to be Destroyed by Severing and Depolymerization in Guard Cells of Open Stomata whereas They Tend to be Retained in Guard Cells of Closed Stomata

It was reported that actin filaments are frequently subject to severing and depolymerization in plant cells [21], and the severing frequency and depolymerization rate of actin filaments should contribute to the construction of certain actin array in cells. We therefore decided to measure the severing frequency and depolymerization rate of actin filaments at different orientations in guard cells of open and closed stomata. We found that, in open stomata, average severing frequency of longitudinally oriented actin filaments reached at 0.07 breaks/μm/s, which was much more frequent than that of actin filaments at other three orientations (open hole columns in Figure 5e, *P* < 0.05; Figure 5a-1). In guard cells of closed stomata, we found that the average severing frequency of longitudinal actin filaments, D-V R actin filaments, V-D R actin filaments, and O actin filaments was determined to be 0.01 breaks/μm/s, 0.02 breaks/μm/s, 0.04 breaks/μm/s, and 0.03 breaks/μm/s (black hole columns in Figure 5e, ND; Figure 5a-2), respectively. The notable difference is that the average severing frequency of longitudinal actin filaments in guard cells of closed stomata is significantly lower than that in guard cells of open stomata (Figure 5e, *P* < 0.05).

We also determined the shrinking rates of actin filaments in guard cells of open and closed stomata. The average shrinking rates of L actin filaments, D-V R actin filaments, V-D R actin filaments and O actin filaments in guard cells of open stomata were determined to be 0.29 μm/s, 0.18 μm/s, 0.19 μm/s, and 0.21 μm/s (open hole columns in Figure 5d), respectively. Among them, L actin filaments shrink faster than actin filaments in other orientations. In guard cells of closed stomata, the average shrinking rates of D-V R actin filaments, V-D R actin filaments, and O actin filaments were determined to be 0.28 μm/s, 0.25 μm/s, and 0.31 μm/s (Figure 5d, D-V R, *P* < 0.01, V-D R, *P* < 0.01, O, *P* < 0.05 between open and closed stomata), respectively. Although there is no overt difference in shrinking rates between actin filaments in different orientations in guard cells of closed stomata, they are higher overall compared to the corresponding parameters in guard cells of open stomata. These data together suggest that longitudinally oriented actin filaments tend to be removed by depolymerization and severing in guard cells of open stomata, whereas they tend to be preserved in guard cells of closed stomata.

### 2.6. Actin Filaments in Different Orientations Have Different Actin Bundling Frequency in Guard Cells of Open and Closed Stomata

The extent of actin bundling impacts the dynamic properties of individual actin filaments and the subsequent construction of actin structures. We therefore decided to determine the bundling frequency for actin filaments in different orientations in guard cells of open and closed stomata. We found that the average bundling frequency of R actin filaments, O actin filaments, and L actin filaments within guard cells of open stomata was determined to be 0.008 events/μm^2^/min, 0.0014 events/μm^2^/min, and 0.0015 events/μm^2^/min (Figure 5f), respectively. This suggests that radially oriented actin filaments have higher actin bundling activity and tend to be stabilized. In guard cells of closed stomata, the average bundling frequency of radially oriented actin filaments, longitudinally oriented actin filaments, and obliquely oriented actin filaments was determined to be 0.004 events/μm^2^/min, 0.016 events/μm^2^/min, and 0.005 events/μm^2^/min (Figure 5f), respectively. Considering the data that the bundling frequency of longitudinal actin filaments is much higher than that of radial actin filaments in guard cells of closed stomata (Figure 5f, *P* < 0.05), it suggests that longitudinal actin filaments tend to be stabilized in guard cells of closed stomata. In addition, the bundling frequency of oblique and longitudinal actin filaments in guard cells of closed stomata was much higher than the corresponding ones in open stomata (Figure 5f, L, *P* < 0.01, O, *P* < 0.05 between open and closed stomata), which might contribute to the transition of radial actin array to longitudinal actin array.

## 3. Discussion

It was reported that actin filaments assume distinct distributions within guard cells from stomata of different apertures [3,4,5,6,7,8,17]. However, how the dynamic behavior of individual actin filaments contributes to the formation and maintenance of distinct actin arrays in guard cells remains largely unknown. Here, we develop a program called GCMA that enables the semiautomatic selection of actin filaments with positional information, which allows us to measure values of angles of all individual actin filaments at once in the guard cell. By tracing the dynamics of individual actin filaments within guard cells from stomata of different apertures, we here provide insights into the formation of radial and longitudinal actin arrays in guard cells of open and closed stomata, respectively.

### 3.1. GCMA is a Useful Tool to Evaluate the Orientations of Individual Guard Cell Actin Filaments

Filamentous structureswere grouped into transverse, longitudinal and oblique array according to the values of angles formed between the filament lines and the long axis of the root in root epidermal cells [22]. The angles formed between actin filaments and the growth axes of pollen tubes were used to evaluate the arrangement of actin filaments in pollen tubes [23,24,25]. Besides, angles of microtubules were determined also between individual microtubules and the long axis of the cells in embryos and hypocotyls of *Arabidopsis* [26,27]. It can be hypothesized that the orientation of actin filaments and microtubules can be evaluated well by measuring the included angle between cytoskeleton line and the long axis of the cell in many types of cells, owing to these cells surrounded symmetrically with the central long axis approximately. However, the shape of guard cell is different from these cells mentioned above, because there is no typical long axis in guard cells and the shape of guard cell is constantly changing with the stomatal movement. Thus, the orientation of guard cell actin cables evaluated by angle values is relevant to the shape of the stomatal pore, and it is necessary to establish an appropriate method for measuring the angle of actin filament in guard cells.

To date, we still lack appropriate approaches to measure the angles of individual actin filaments in guard cells automatically in large scale. In a previous study, Higaki et al. developed an image analysis framework that is able to measure the mean angular differences of the digital images between actin filament pixel pairs and the nearest pixel pairs of the stomatal pore edges and then get a radial factor reflecting the overall actin pattern within guard cells from particular stomata [5]. It is very effective for evaluating the overall actin pattern in digital images of particular stomata. However, it fails to measure all individual actin filaments to yield the values of the angles at one time. Therefore, development of a method to automatically perform one-off measurement of the angles of all actin filaments in guard cells is important. When we analyze the dynamic parameters of guard cell actin filaments, it is necessary to determine the orientation of single actin filament evaluated by angle value firstly. Toward solving this problem, in this paper, we use the two ending points of the simulated actin filament under image processing using MATLAB to determine the actin filament line, and find the nearest point on stomatal pore edge that shows the nearest distance from the actin filament line or its extending line, then use this nearest point to determine the relative radial line of the actin filament concerning to the stomatal pore edge. Last, get the included angle between the actin filament line and its relative radial line as the angle of the actin filament, so that each guard cell actin filament under the unique stomatal aperture can obtain a unique angle value. Besides, our method can measure angle values of several individual actin filaments once in a guard cell, at the same time get the length values of single actin filaments in a guard cell and the width/length ratio to quantify the stomatal aperture. According to the values of angles measured by GCMA program, all of guard cell actin filaments can be explicitly distinguished into three groups, radial, oblique, and longitudinal orientation in guard cells (Figure 2). This provides an important premise to study different dynamic characteristics on all of three differently oriented actin filaments in guard cells, further providing better opportunities to illustrate the detailed function of each of actin binding proteins working in guard cell system in the near future.

### 3.2. The Majority of De Novo Nucleated Actin Filaments Grow Radially and Longitudinally in Open and Closed Stomata, Respectively

As actin filaments are arrayed into distinct structures that regulate stomatal aperture [28], visualization of the dynamics of actin filaments in guard cells is helpful to understand the mechanism of the formation of the specific actin arrays, and the regulation of stomatal movement. From tracking the dynamics of the formation of single actin filaments in open stomata, about half of the newly formed guard cell actin filaments elongated along a radial orientation (Figure 3). It included large parts of actin filaments which nucleated from the dorsal side and elongated along a dorsal-to-ventral radial orientation and less parts of actin filaments which nucleated from the ventral side and elongated along a ventral-to-dorsal radial orientation. Both of these two parts of newly formed guard cell actin filaments are helpful to form the radial actin array in open stomata. In contrast, the proportion of the radially oriented actin filaments decreased from about one-half to one-third in all of the newly formed guard cell actin filaments whereas the proportion of the longitudinally oriented actin filaments increased from approximately one-third to nearly one-half. It is helpful to form the longitudinal actin array in closed stomata. No matter radially oriented actin filaments or longitudinally oriented actin filaments, the proportion of nucleation occurred in dorsal side were more than that in ventral side, and this might because that the area of dorsal region is bigger than the area of the ventral region. However, the nucleation frequency per unit area between dorsal region and ventral region seemed no difference (Figure 3 and Figure 4). Whether these actin filaments are nucleated by the Formins and/or ARP2/3 complex needs to be determined in the future. Functional analysis of Formins- and ARP2/3 complex-deficient mutants as well as observing the effect of pharmacological treatments with Arp2/3 and formin inhibitors on actin dynamics in guard cells likely provide insights into this question in the future.

### 3.3. Radially Oriented Actin Cables in Open Stomata whereas Longitudinally Oriented Actin Cables in Closed Stomata Tend to be Preserved

In open stomata, the majority of the newly formed actin filaments elongate along the radial orientation and they are rarely subject to severing and depolymerization and therefore they tend to be preserved. Meanwhile, we found that the newly formed actin filaments that elongate longitudinally tend to be destroyed by severing and depolymerization in open stomata. The combination of the dynamic behaviors of actin filaments contributes to the formation of radial actin array in guard cells of open stomata. However, we found that the newly formed actin filaments in longitudinal orientation elongate faster, bundle more frequently, and are less frequently subject to severing when compared to actin filaments in other orientations in guard cells from closed stomata. Consequently, actin filaments are arranged into longitudinal actin array in guard cells of closed stomata. As actin-binding proteins (ABPs) are direct regulators of actin dynamics, differential dynamic properties of actin filaments within guard cells of stomata at different apertures suggest that those ABPs are differentially regulated in stomata at different stages. In consideration of actin filaments in different orientations that have differential severing frequency, the activity of actin severing proteins (like ADF and villin) might be differentially regulated in stomata at different stages. Therefore, differential regulation of those severing proteins likely facilitates the construction of different actin arrays. Indeed, ADF and villin, were predicted to facilitate the organization of actin filaments in pollen tubes [23,29]. Certainly, besides ADFs [4,8,10] and villins, some other actin-binding proteins might also be involved in this actin restructuring and maintaining process. Careful documentation of the role of those ABPs in guard cells will enrich our understanding of the mechanism underlying the construction and maintenance of different actin arrays in guard cells in the future.

## 4. Materials and Methods

### 4.1. Plant Materials and Growth Conditions

GFP-ABD2-GFP transgene lines under the background of Arabidopsis (*Arabidopsis thaliana*) ecotype Columbia-0 (Col-0) [30] were grown in a greenhouse under 16-h-light/8-h-dark cycle. The plants grew at 18–22 °C, without any water-deficit stress. Fully expanded leaves from 3- to 4-week-old plants with the leaf size of approximately 8–10 mm width and 10–15 mm length were used for stomatal aperture measurements and observation of the organization and dynamics of actin filaments in guard cells.

### 4.2. Stomatal Bioassay and Visualization of Actin Filaments by Confocal Laser Scanning Microscopy (CLSM)

For studying the relationship between orientations of single actin filaments and stomatal apertures in stomatal closure under diurnal cycle, we harvested rosette leaves of *Arabidopsis* ecotype Col-0 marked by GFP-ABD2-GFP at 12:00 p.m., 3:00 p.m., 6:00 p.m., and 9:00 p.m. Then the intact leaves were observed under the microscope, and stomata in the abaxial epidermis were determined. GFP-ABD2-GFP marked guard cell actin filaments were scanned in a 0.5 μm-internal z-stack layer under the Olympus FV1000MPE multiphoton confocal laser scanning microscope equipped with a ×100 oil objective (numerical aperture 1.4). In order to perform dual visualization of actin filaments and guard cells, we scanned actin filaments by the excitation of a 488 nm argon laser and an emission of 505–545 nm, and scanned guard cells by the bright field illumination. The maximal intensity projection images were generated with the Image J Software (version 1.46g). The stomata were classified into four stages according to the ratio of width/length of stomatal pore. Angle values of single actin filaments were measured in each of guard cells of four stages. The experiment repeats three times.

### 4.3. Measurement of Angles of Actin Filaments in Guard Cells

The angles of actin filaments were measured with GCMA program built in MATLAB software (see the installed method in Supplemental Materials). Firstly, we get the center point O^o^ of the simulated circle through the right stomatal pore edge, and the point O’ of stomatal pore edge which owned the nearest distance to the filament line or the extension line of the filament. Then the relative radial line of the filament is got through the two-point connection of point O^o^ and O’. Last the included angle between the filament or its extension line and its radial line is got as the angle of this filament (the detailed schematic diagram of the measurements can be found in Supplemental Figure S3). The kernel density of angle distribution of guard cell actin filaments was processed using R programming language (available online: http://www. R-project.org; version 2.15.1) as previously described [29]. Then all of actin filaments were classified into radial, oblique, and longitudinal orientation according to the angle values of single actin filaments in each of four stages (0 ≤ angle value < 40, radial; 40 ≤ angle value < 90, oblique; angle value = 90, longitudinal). The results were the average in three repeats ± SE. At least 600 actin cables from 40 guard cells were selected for the measurement.

### 4.4. Visualization of the Dynamics of Actin Filaments in Guard Cells by Total Internal Reflection Fluorescence Microscopy (TIRFM)

Actin filaments were decorated with GFP-ABD2-GFP in guard cells as described previously [30]. The intact leaves were cut from the transgenic plants expressing GFP-ABD2-GFP at 12:00–2:00 p.m. for open stomata and 6:00–8:00 p.m. for closed stomata. Guard cells from abaxial epidermis in the intact leave were observed under an inverted Olympus IX81 microscope equipped with a ×100 oil objective (numerical aperture 1.49) by TIRF illumination with the excitation wavelength set at 488 nm and the emission wavelength offset at 505–545 nm in the dark. The time-lapse images were captured at 2 s intervals for 10 min with a Photometrics Evolve 512 Delta EMCCD camera (Photometrics, Tucson, USA) driven by MicroManager software.

### 4.5. Quantitative Analyses of the Dynamics of Actin Filaments in Guard Cells

Time-lapse images of actin filaments in guard cells were processed with Image J software. To quantify the percentage of the newly formed actin filaments elongating in different orientations, we analyzed 200–400 actin filaments from at least 20 guard cells for open stomata and closed stomata, respectively. To quantify the nucleation frequency of actin filaments within different regions of guard cells, we initially divided guard cells into two parts, the dorsal region and ventral region, by means of a central boundary of the guard cell longitudinally which distanced the same to the dorsal wall and the ventral wall of the guard cell (see dotted pink lines drawn in Figure 3b and Figure 4b). Then we measured the area of dorsal region and ventral region of the guard cell. The nucleation frequency was defined as the number of actin nucleation events per area of region per unit time (nucleation events/μm^2^/min). At least 20 guard cells from three experimental repeats were analyzed for each group. Other parameters—such as elongation rate, shrinkage rate, severing frequency and bundling frequency of actin filaments—were calculated as previously described [21,29,31]. 20–60 actin filaments from at least 20 guard cells from three experimental repeats for each group were measured.

## 5. Conclusions

Here, we divided stomata into four distinct stages based on their apertures (width/length) and found that actin filaments are arrayed into distinct structures within guard cells of stomata at different stages. Given that guard cells have unique geometry and undergo changes during stomatal closure and opening, to distinguish individual actin filaments at different orientations, we developed the GCMA program, which enables the measurement of the angles of all actin filaments with positional information at once in guard cells at different stages of stomatal movement. Aided by GCMA, we traced the dynamics of individual actin filaments and measured the parameters associated with their dynamics. We found that de novo nucleation of actin filaments occurs at both dorsal and ventral sides of guard cells in open and closed stomata, whereas the majority of newly nucleated actin filaments elongate in radial and longitudinal directions in guard cells of open and closed stomata, respectively. In addition, we found that radial actin filaments tend to form actin bundles whereas longitudinal actin filaments tend to be removed by severing and depolymerization in guard cells of open stomata. By contrast, longitudinal actin filaments tend to form actin bundles that are less frequently subject to severing in guard cells of closed stomata. These observations provide insights into the formation and maintenance of distinct actin arrays in guard cells of stomata at different stages. The developed GCMA program here will facilitate the studies on the regulation of the organization and dynamics of actin filaments in guard cells during stomatal movement in the future.

## Supplementary Materials

Supplementary materials can be found at https://www.mdpi.com/1422-0067/20/11/2753/s1.

## Abbreviations

GCMA	Guard Cell Microfilaments Analyzer
MATLAB	Matrix Laboratory
R	Radial
O	Oblique
L	Longitudinal
D-V R	Dorsal-to-Ventral Radial
V-D R	Ventral-to-Dorsal Radial
CLSM	Confocal Laser Scanning Microscopy
TIRFM	Total Internal Reflection Fluorescence Microscopy
