# Supplementary material for: Guard Cell Microfilament Analyzer Facilitates the Analysis of the Organization and Dynamics of Actin Filaments in Arabidopsis Guard Cells
Source: Int J Mol Sci. 2019 Jun 5;20(11):2753. doi: 10.3390/ijms20112753 (PMC6600335; doi:10.3390/ijms20112753)
Supplement: Supplementary file 1 [file ijms-20-02753-s001.zip › ijms-505258-SI.pdf]

Guard Cell Microfilament Analyzer Facilitates the Analysis of the Organization and Dynamics of Actin Filaments in *Arabidopsis* Guard Cells

Xin Li, Min Diao, Yanan Zhang, Guanlin Chen, Shanjin Huang, Naizhi Chen

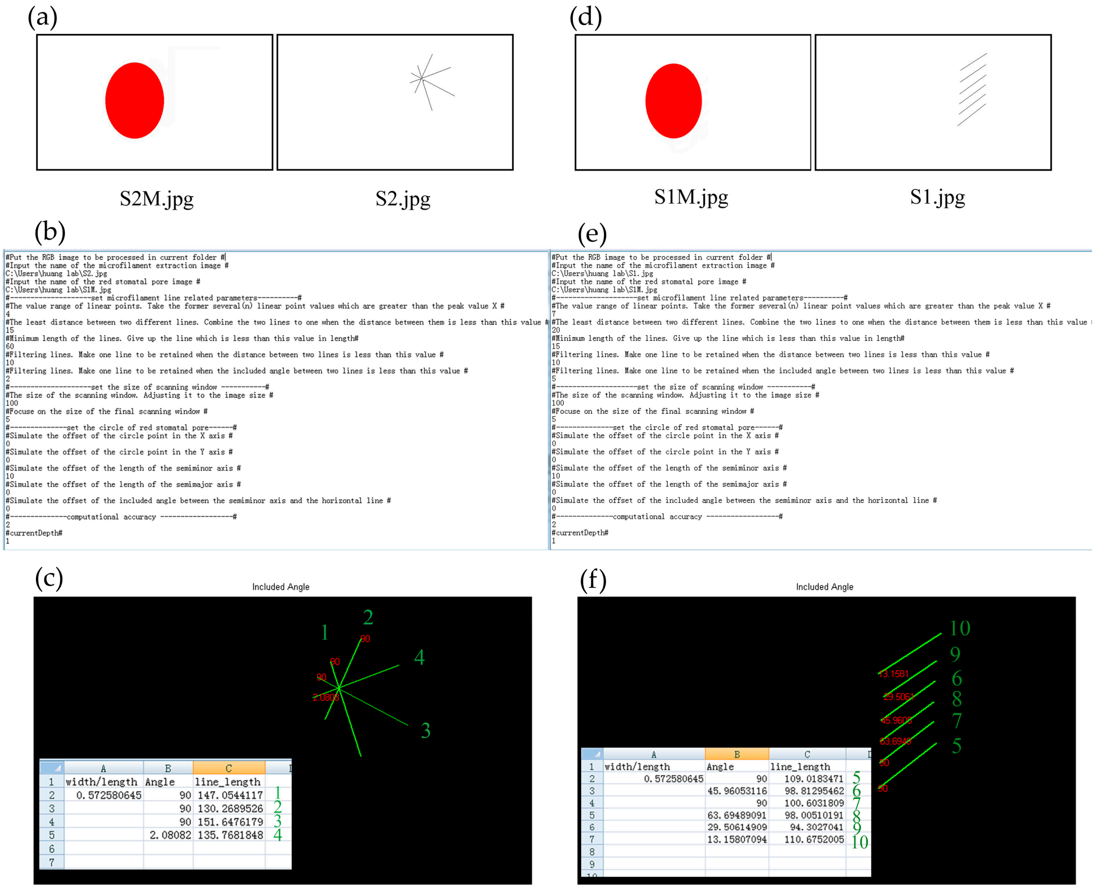

Figure S1. Procedures for angular evaluations of individual filaments by GCMA program. The framework introduced in this study can be divided into three major steps, including inputting the image, adjusting the parameters to recognize the filaments and the stomatal pore well in the interface of the GCMA program and collecting the output data. (**a**)(**d**) The simulated images for inputting into program. Stomatal pore and guard cell actin filaments in the right side of stomata were divided from the raw fluorescent image of guard cell actin cytoskeleton. (**b**)(**e**) Interface of the GCMA program. Various modifiable parameters can be adjusted to make the filaments and stomatal pore to be recognized well. The detailed description of the parameters in each of lines can be seen in Supplemental PPT. (**c**)(**f**) Running GCMA.exe and last the results of angle values of each of the filaments can be output. The green lines represent the simulated filaments for image in (**a**) and (**d**), and the red numbers represent the angle values of the simulated filaments. Table in the left showed the output result of Excel which contained angle value, length value of each of filaments and

width/length value of the stomatal pore. The green numbers marked behind each of line in Excel represent each of filaments which marked with the same green number in the right. The installation procedure of GCMA program and the detailed example on how to use the GCMA program can be found in Supplemental PPT.

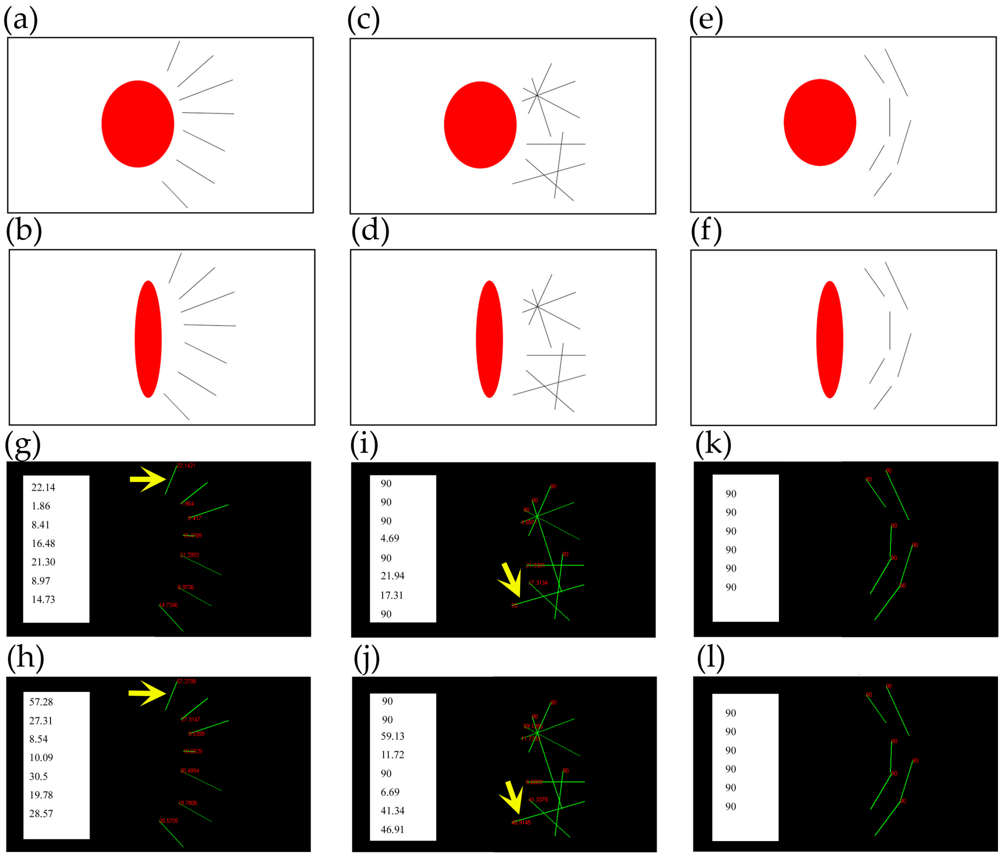

Figure S2. Different angle values of guard cell microfilaments in open stomata and closed stomata. (**a**)(**b**), (**c**)(**d**), (**e**)(**f**) represent three types of microfilament arrangements in open and closed stomata, respectively. (**g**)(**h**), (**i**)(**j**), (**k**)(**l**) represent the angle value results of each of filaments corresponding to (**a**)(**b**), (**c**)(**d**), (**e**)(**f**), respectively. The green lines represent the simulated filaments, and the red numbers represent the angle values of the simulated filaments. The numbers in the white area are the magnified angle values corresponding to the red numbers in a top-down arrangement. Yellow arrows indicate the different angle values of the same type of filaments in open and closed stomata, reflecting the different actin arrays.

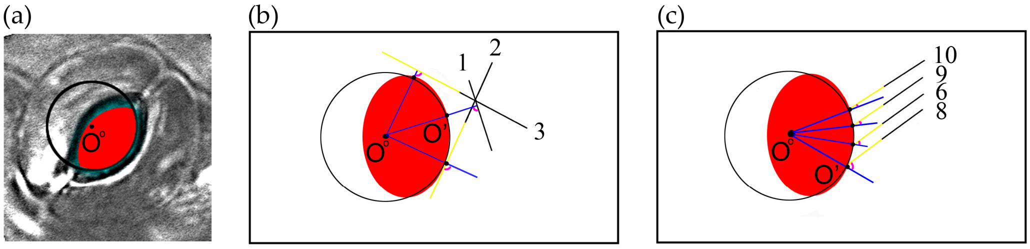

Figure S3. Schematic diagram of angle measurement in individual guard cell actin filaments. (**a**) Simulate the circle through stomatal pore edge of the right guard cell of stomata and the center point of the simulated circle (O^o). (**b**) If the lines or the extension of the lines are not cross with the stomatal pore edge, chose the point (O′ indicated the black dot) on stomatal pore edge which owned the shortest distance with the line (line 1), and make the connection of point O^o and O′ to determine the radial line (blue line), then measure the included angle between the line and this radial line. (**c**) If lines or the extension of the lines cross with the stomatal pore edge, chose the crossed point (O′ indicated the black dot of line 8) on stomatal pore edge, and make the connection of point O^o and O′ to determine the radial line (blue line), then measure the included angle between the filament line (or yellow line) and this blue line.

O′ represent the point on stomatal pore edge owned the shortest distance to the line or the extension of the line and O^o represent the centre of the simulated circle. The included angles (purple marked angles) between the filament lines (or yellow lines) and their corresponding radial (blue) lines were measured. Line 1, 2, 3 in (**b**) and line 6, 8, 9, 10 in (**c**) were chosen from Supplemental Figure 1**c** and Supplemental Figure 1**f**, which were marked by green numbers. Yellow lines showed the extension of the filament lines, blue lines showed the radially oriented lines passing through the specific points on stomatal pore edge. When the width/length ratio is small enough, it is difficult to obtain the corresponding simulated circle. At this time, stomatal pore edge and the long axis of stomatal pore are nearly overlapped, so angle value is defined as the included angle between the line and the long axis of stomatal pore.

Movie 1. Dynamics of actin nucleation and elongation in open stomata

Movie 2. Dynamics of actin nucleation and elongation in closed stomata

Movie 3. Longitudinally oriented actin filament was severed more frequent than radially oriented filament in open stomata

Movie 4. Severing events of radially and longitudinally oriented actin filament in closed stomata.

Movie 5. Formation of radial actin bundle in open stomata

Movie 6. Formation of longitudinal actin bundle in closed stomata

GCMA_pkg. Installation package of GCMA program, including the sample images.

Main.conf. Running interface of the GCMA.exe

PPT. Usage of the GCMA program
