# Supplementary material for: Guard Cell Microfilament Analyzer Facilitates the Analysis of the Organization and Dynamics of Actin Filaments in Arabidopsis Guard Cells
Source: Int J Mol Sci. 2019 Jun 5;20(11):2753. doi: 10.3390/ijms20112753 (PMC6600335; doi:10.3390/ijms20112753)
Supplement: Supplementary file 1 [file ijms-20-02753-s001.zip › PPT.pdf]

1 Program Installation and Run

1 Open GCMA_pkg and then install the MCRinstaller.exe file (32-bit operating system) in the package.

2 Open the GCMA.exe file and find out its installation folder according to the installation route in DOS interface.

```
C:\Users\HUANGL~1\AppData\Local\Temp\huang lab\mcrCache7.16\GCMA_00\130928\To Li
\To Li-1005/main.conf
```

3 Manually put the main.conf file in GCMA installation package into the installation folder mentioned above.

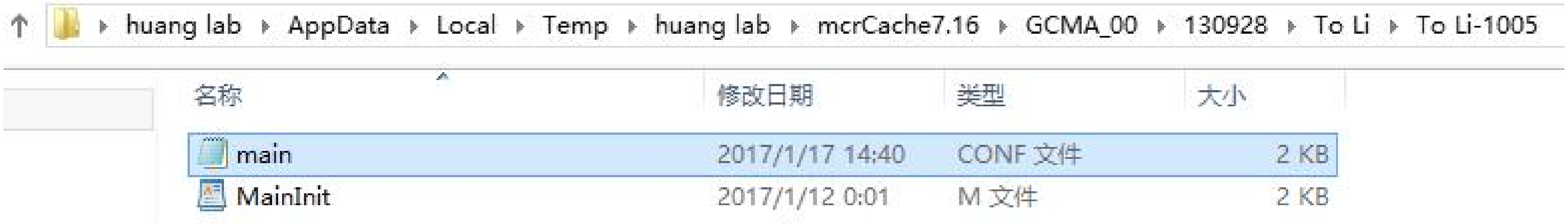

4 Open main.conf file and change parameters in it, then save main.conf and run the program of GCMA.exe to output the result.

Parameter Interface (main.conf file)

represents the parameters that can be changed

Line

```
#Put the RGB image to be processed in current folder #
#Input the name of the microfilament extraction image #
(3) C:\Users\huang lab\SYT.jpg
#Input the name of the red stomatal pore image #
(5) C:\Users\huang lab\SYT-M.jpg
#--------------------set microfilament line related parameters----------#
#The value range of linear points. Take the former several(n) linear point values which are greater than the peak value X #
(8) 110
#The least distance between two different lines. Combine the two lines to one when the distance between them is less than this value #
(10) 3
#Minimum length of the lines. Give up the line which is less than this value in length#
(12) 10
#Filtering lines. Make one line to be retained when the distance between two lines is less than this value #
(14) 5
#Filtering lines. Make one line to be retained when the included angle between two lines is less than this value #
(16) 5
#--------------------set the size of scanning window -----------#
#The size of the scanning window. Adjusting it to the image size #
(19) 100
#Focuse on the size of the final scanning window #
(21) 5
#--------------set the circle of red stomatal pore------#
#Simulate the offset of the circle point in the X axis #
(24) 0
#Simulate the offset of the circle point in the Y axis #
(26) 0
#Simulate the offset of the length of the semiminor axis #
(28) 20
#Simulate the offset of the length of the semimajor axis #
(30) 0
#Simulate the offset of the included angle between the semiminor axis and the horizontal line #
(32) 0
#--------------computational accuracy -----------------#
2
#currentDepth#
1
```

2 Input the Image

```
#Put the RGB image to be processed in current folder #
#Input the name of the microfilament extraction image #
(3) C:\Users\huang lab\L1-1.jpg
#Input the name of the red stomatal pore image #
(5) C:\Users\huang lab\L1M.jpg
```

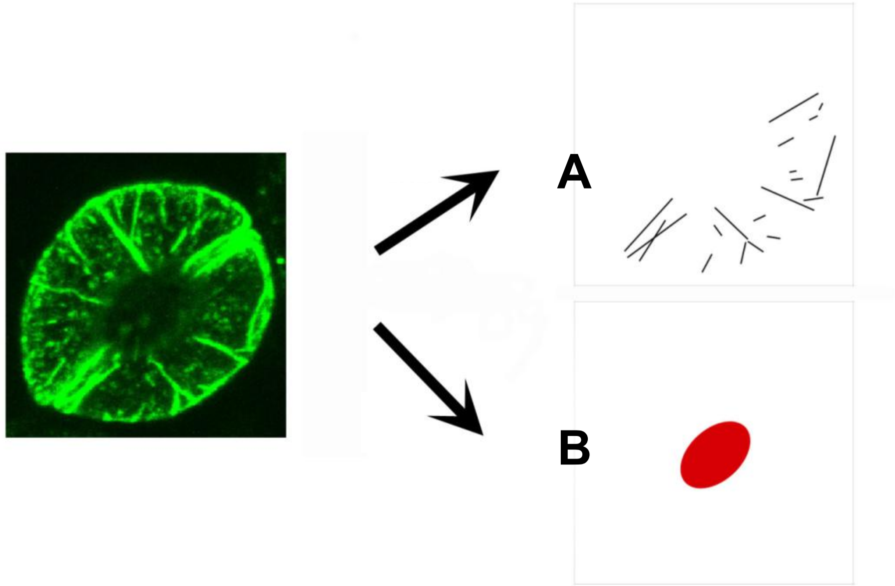

A Image named L1-1

B Image named L1M

(3)(5) Correct the route that the images of guard cell actin filament **(A)** and stomatal pore **(B)** existed.

(A) and **(B)** is extracted in the right half of stomata through “skeletonizing” (Higaki *et al*., 2010) or drawing in Photoshop software.Then flip the fluorescent image horizontally, extract the other guard cell actin filaments and stomatal pore images in the left half of stomata of original image.)

3 Adjust Parameters

3-1 To make the simulated filaments (Figure F in next slide) similar to the raw image (Figure C in next slide) in the largest extent

```
     #--------------------set microfilament line related parameters----------#
     #The value range of linear points. Take the former several(n) linear point values which are greater than the peak value X #
(8)  110
     #The least distance between two different lines. Combine the two lines to one when the distance between them is less than this value #
(10) 3
     #Minimum length of the lines. Give up the line which is less than this value in length#
(12) 8
     #Filtering lines. Make one line to be retained when the distance between two lines is less than this value #
(14) 6
     #Filtering lines. Make one line to be retained when the included angle between two lines is less than this value #
(16) 5
```

(8) If the number of simulated lines was less than it in the raw image, increase this value; otherwise, decrease this value.

(10) The larger the value, the longer lines is simulated; the smaller the value, the shorter lines is simulated.

(12) The bigger the value, the less the number of lines detected; the smaller the value, the more the number of lines detected.

(14) Only one of two lines with spacing less than this value is left.

(16) Keep one of two straight lines with angle less than this value.

Image Simulation Results

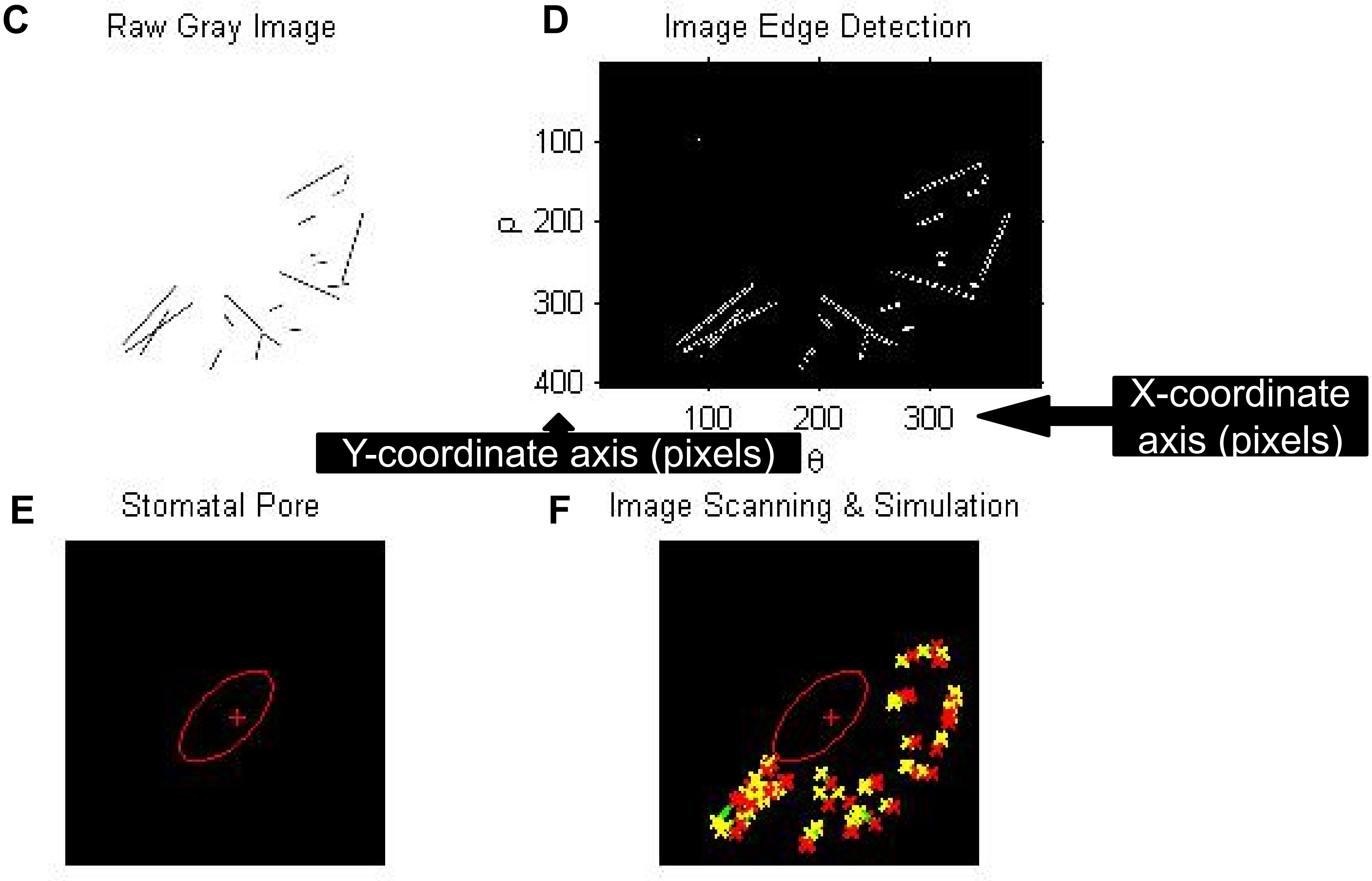

C-F Output result of image detction

3 Adjust Parameters

3-2 To make the simulated stomatal pore (Fig.E in last slide) similar to the raw image (Fig.B) in the largest extent

```
     #--------------------set the size of scanning window -----------#
     #The size of the scanning window. Adjusting it to the image size #
(19) 100
     #Focused on the size of the final scanning window #
(21) 5
```

(19) Stomatal pore region can be determined according to the scale axis (pixels) in Figure D

(21) Do not change

```
     #-------------set the circle of red stomatal pore------#
     #Simulate the offset of the circle point in the X axis #
(24) 0
     #Simulate the offset of the circle point in the Y axis #
(26) 0
     #Simulate the offset of the length of the semiminor axis #
(28) 0
     #Simulate the offset of the length of the semimajor axis #
(30) 20
     #Simulate the offset of the included angle between the semiminor axis and the horizontal line #
(32) 20
```

Continued the previous slice

(24) If you want the simulated stomatal pore to move left, decrease this value; otherwise increase this value.

(26) If you want the simulated stomatal pore to move up, decrease this value; otherwise increase this value.

(28) If you want to increase the width of the simulated stomatal pore, increase this value; otherwise decrease this value.

(30) If you want to increase the length of the simulated stomatal pore, increase this value; otherwise decrease this value.

(32) If you want the stomatal pore to be rotated clockwise (G→H→I), increase this value; otherwise decrease this value.

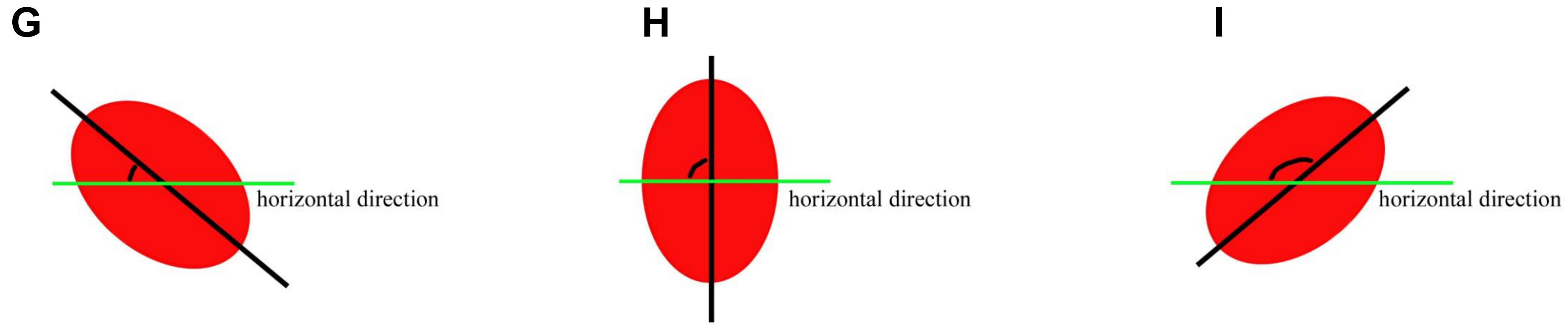

(G,H,I) Schematic view of the included angle between the long axis (black line) of the stomatal pore and the horizontal line (green line) when stomatal pore located in three different positions.

4 Save the Parameters Changed, Run the Program, and Output the Result

J

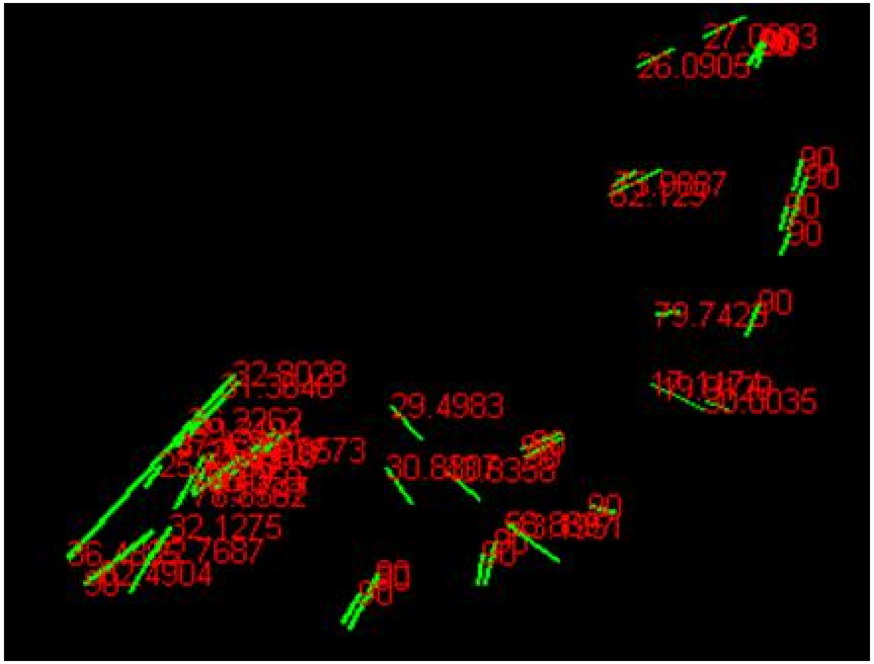

J Image simulation results

Green lines indicate the recognized lines, red numbers indicate the corresponding angle values of the lines.

K

	A	B	C
1	width/length	Angle	line_length
2	0.479452055	32.8027808	99.82484661
3		90	31.40063694
4		73.98871865	9.433981132
5		36.48847008	36.06937759
6		37.42265065	36.06937759
7		26.68271606	17.49285568
8		73.74698429	12.04159458
9		71.05729172	12.04159458
10		90	22.20360331
11		31.36482861	21.26029163
12		82.49037806	27.01851217
13		78.85615405	27.01851217
14		43.19510868	9.219544457
15		43.83580775	18.43908891
16		29.49834727	17.69180601
17		90	22.13594362
18		47.12317579	9.219544457
19		22.82175003	24.18677324
20		22.76873585	18.35755975
21		90	11.66190379
22		90	9.433981132
23		90	8.544003745
24		90	12.36931688
25		90	21.9544984
26		62.12897445	22.36067977
27		90	17.4642492
28		90	24.59674775
29		58.88972011	25.8069758
30		19.91289368	18.78829423
31		90	16.15549442
32		90	8.94427191
33		32.12751091	18.02775638
34		90	13.03840481
35		17.40584645	11.18033989
36		26.22618958	11.40175425
37		25.62050095	10
38		39.31914181	18.38477631
39		90	9.486832981
40		90	8.544003745
41		90	13.92838828
42		90	10.19803903
43		90	12.16552506
44		90	15.65247584
45		90	9.219544457
46		26.09054068	15.65247584
47		27.00834693	17.88854382
48		79.74233772	9.055385138
49		90	9.433981132
50		17.11737049	15.65247584
51		30.00350372	11.70469991
⋮		⋮	⋮

K Table results

Open the test.xls in the same folder with GCMA.exe, and width/length value of the stomatal pore, angles and the lengths of each of lines can be seen in the table.

Lastly, select and record all of angle values of the accurately simulated lines, then remove test.exel file in order to create the new test.xls file in the next program running. (If the simulated image can not be simulated well with the original image, change the parameters in the main.conf file then continue the procedure described above.)

5 Probable Problems and Solutions

If filaments (for example in **Figure L**) are too complex to be detected well, segment the image to three small parts (for example in **Figure M,N,O**) or more manually, input each of images and measure the angles of lines in each part, separately.

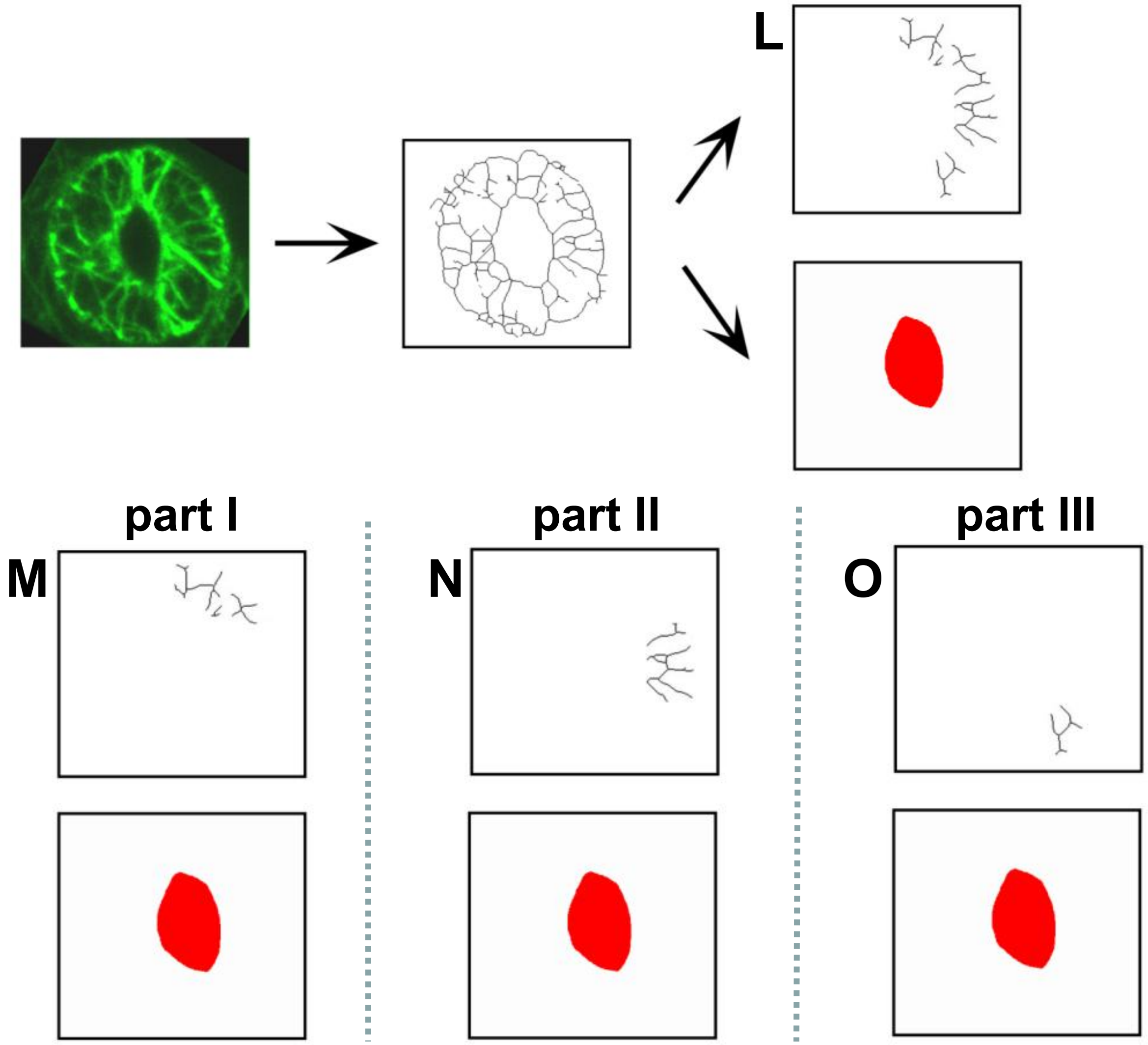

L Filaments extraction

M,N,O Filaments extraction in small parts segmented from Figure L

Output result of part I

P

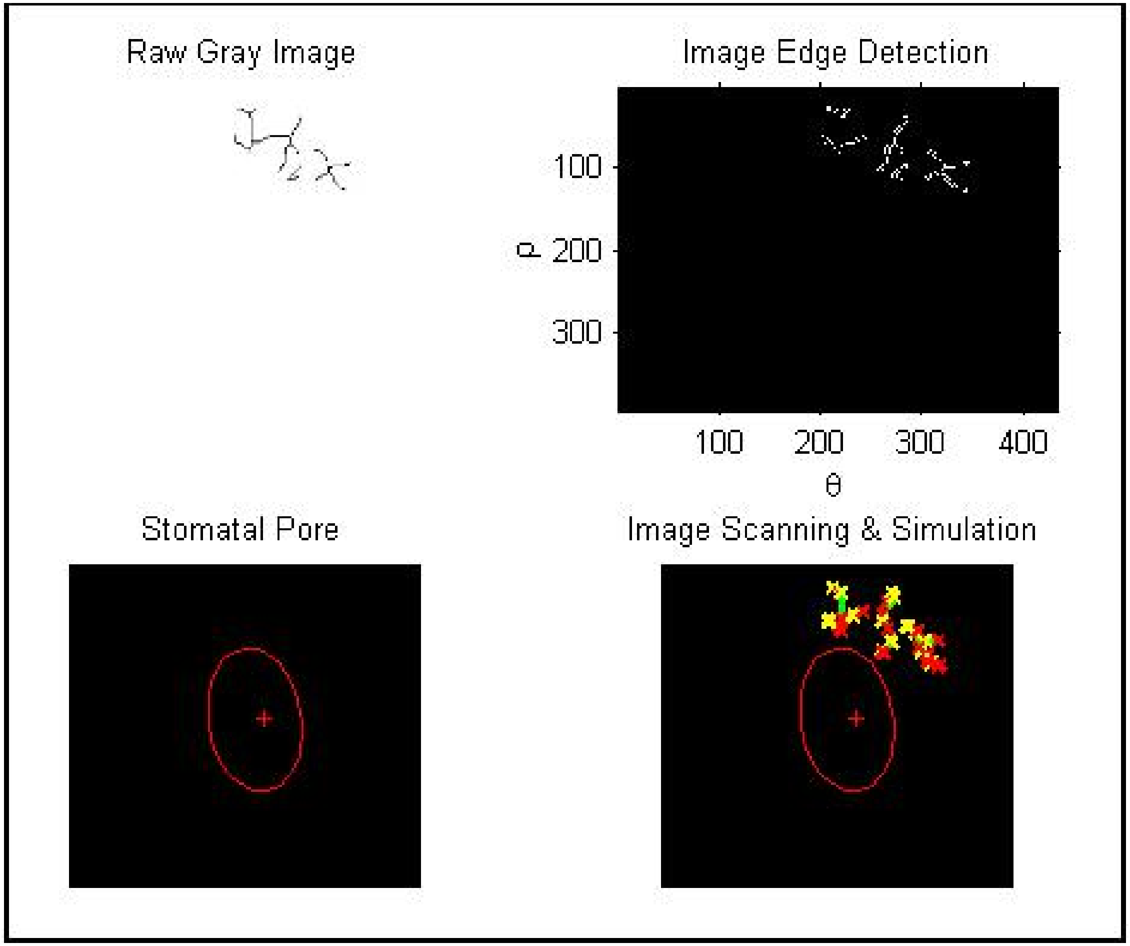

Q

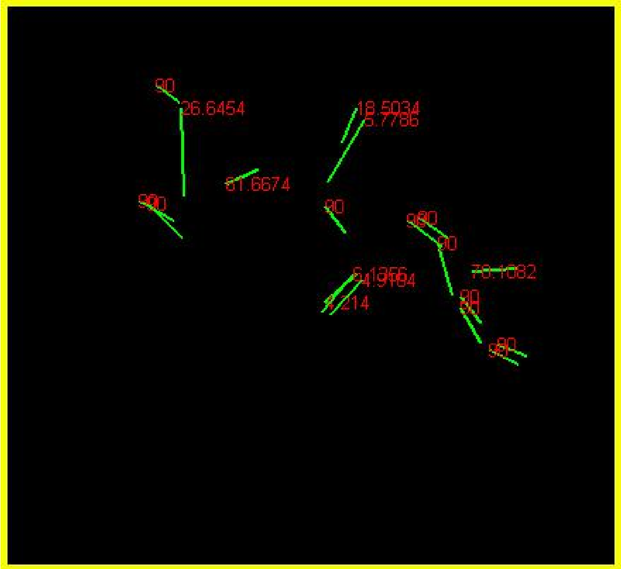

R

	A	B	C
1	width/length	Angle	line_length
2	0.647727273	90	10
3		21.98018	31.01612484
4		14.52919	13
5		90	10.77032961
6		90	11.18033989
7		1.874187	25.55386468
8		76.11975	16.03121954
9		90	11.40175425
10		7.350573	14.86606875
11		90	13.89244399
12		1.769875	16.2788206
13		90	15
14		90	18.68154169
15		90	12.08304597
16		2.877158	17.02938637
17		90	13.89244399
18		90	16.97056275
19		90	11.40175425
20		90	12.20655562
21			

P Image detection result of image M
Q Image simulation result of image M
R Table result of image M

Output result of part II

T

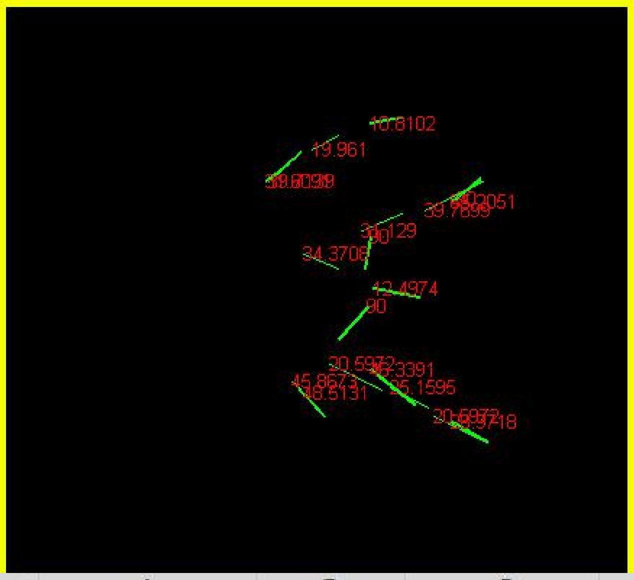

S

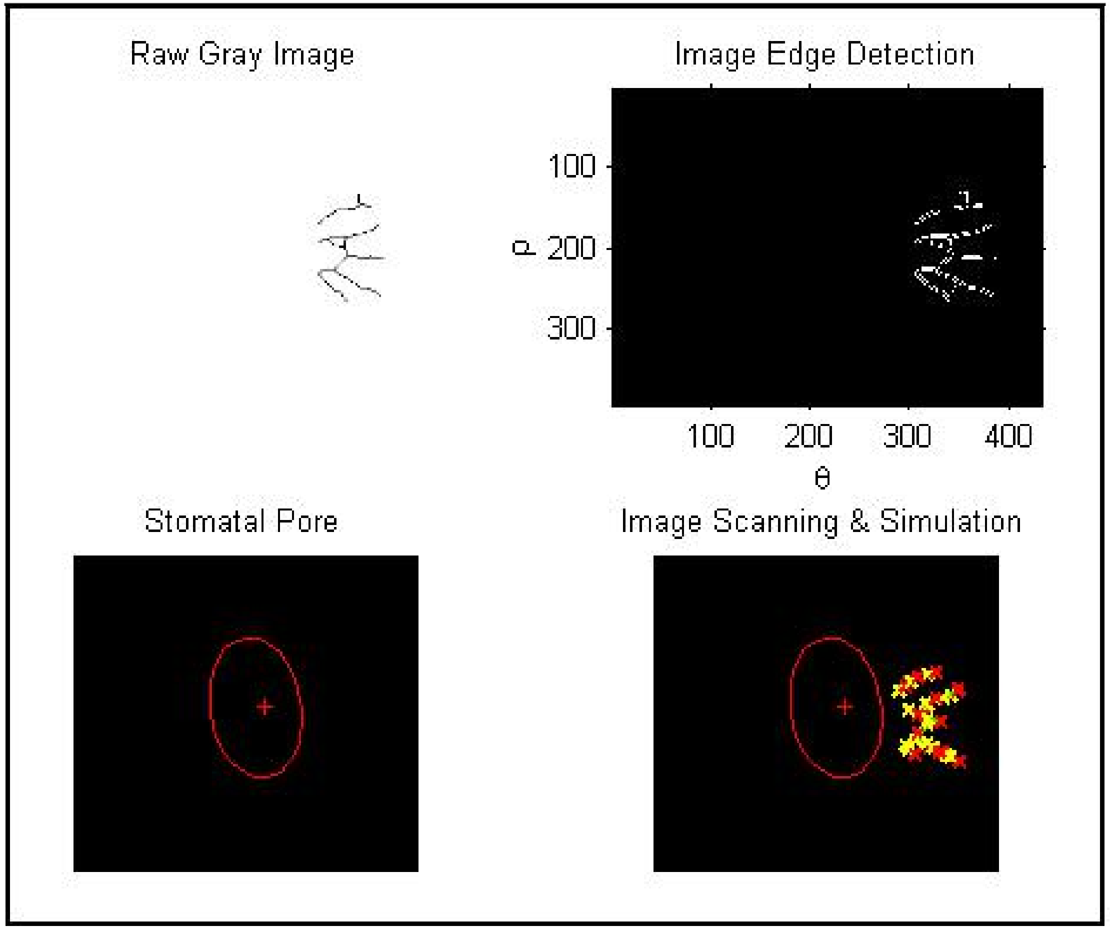

U

	A	B	C
1	width/length	Angle	line_length
2	0.647727273	18.18153	20.1246118
3		90	10.63014581
4		18.18153	20.1246118
5		22.71552	14.76482306
6		26.44657	13.89244399
7		90	11.18033989
8		11.39259	11.18033989
9		90	14.86606875
10		64.78612	12.20655562
11		32.61865	13
12		42.03958	16.2788206
13		39.65669	22.36067977
14		37.8998	14.86606875
15		31.03808	15.23154621
16		43.19279	19.20937271
17		30.14911	10
18		18.87689	10.29563014
19		11.18594	16.2788206
20		44.52007	10.63014581
21			
22			

S Image detection result of image N
T Image simulation result of image N
U Table result of image N

Output result of part III

V

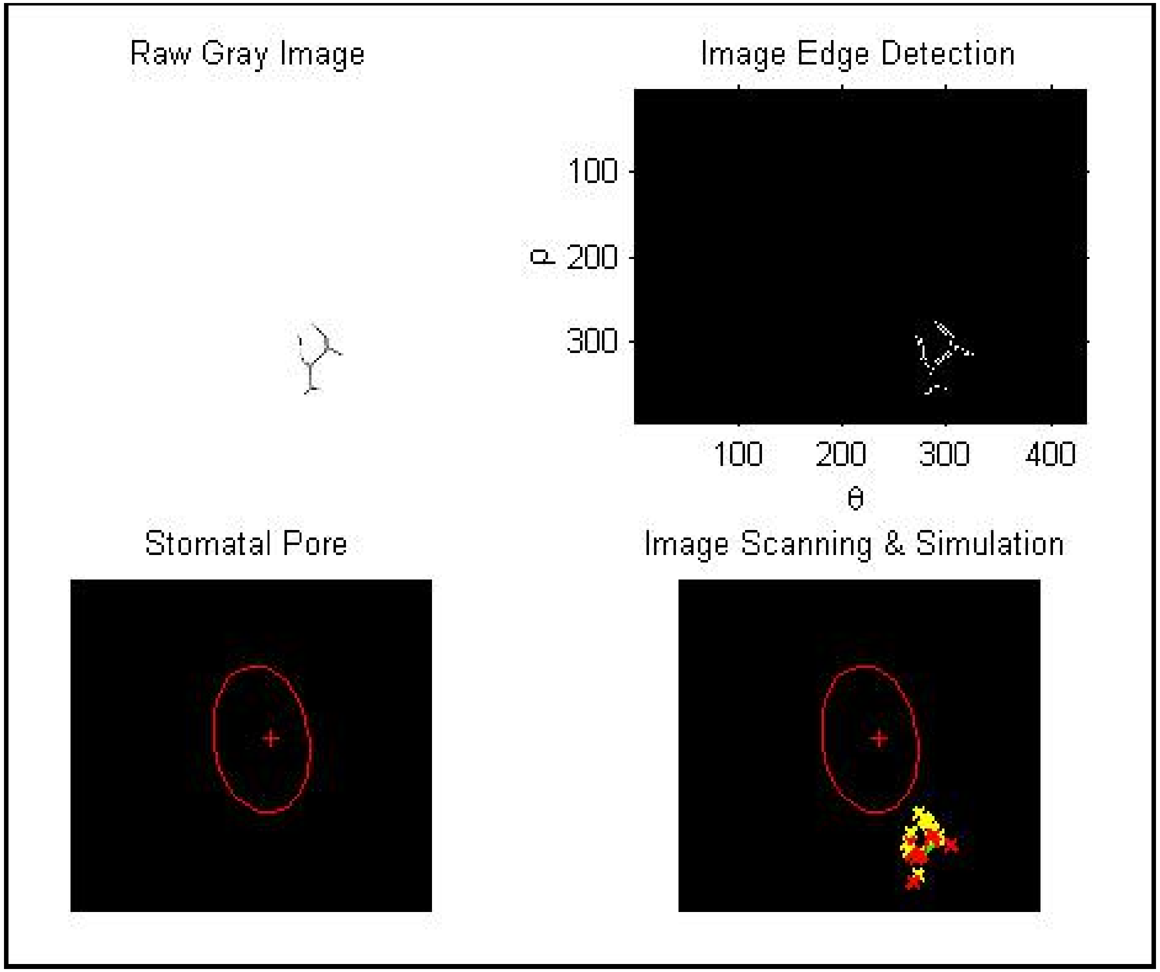

W

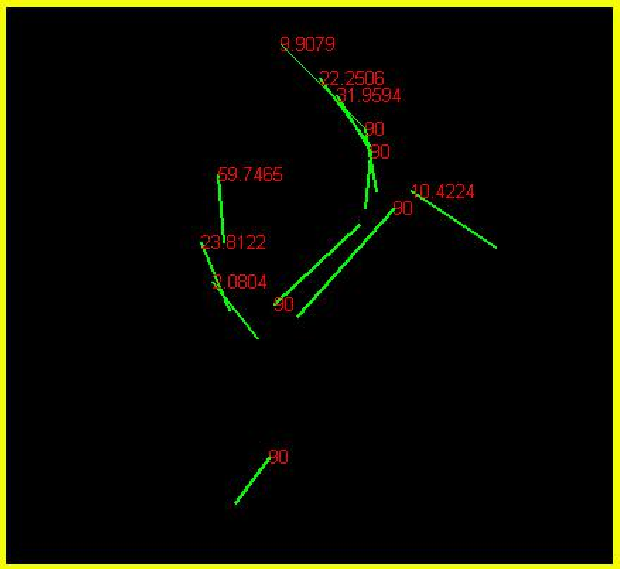

X

	A	B	C
1	width/length	Angle	line_length
2	0.647727273	5.939916	21.21320344
3		90	20.51828453
4		90	25.49509757
5		18.16123	15
6		19.24406	13
7		53.75687	12.04159458
8		27.68344	11.66190379
9		14.5943	18.02775638
10		90	10.04987562
11		6.846409	12.80624847
12		90	10
13		75.70237	11.18033989
14			

V Image detection result of image O

W Image simulation result of image O

X Table result of image O

Result comparison

Compared to the results of all filaments in a guard cell measured at one time, filaments segmented into small parts can be detected and measured precisely.

Y

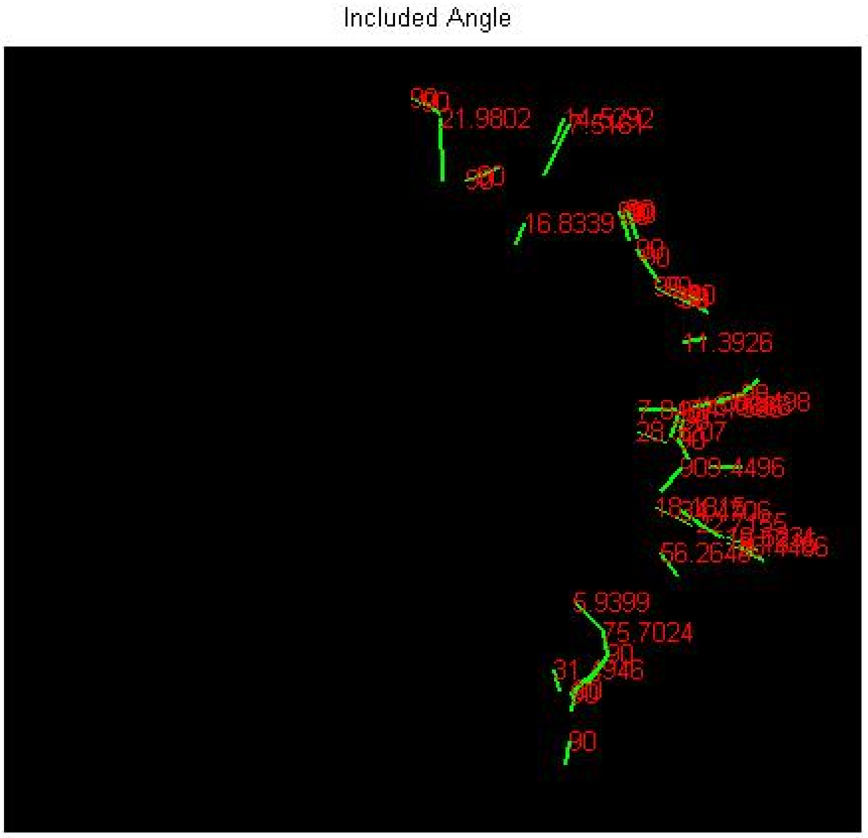

Z

	A	B	C	D
1	width/length	Angle	line_length	
2	0.647727273	90	10	
3		90	12.64911064	
4		18.1815264	20.1246118	
5		90	11.18033989	
6		90	10.77032961	
7		18.0665582	21.84032967	
8		90	10.63014581	
9		90	17.4642492	
10		10.6230666	16.15549442	
11		90	13.03840481	
12		90	10.19803903	
13		31.4946125	10.44030651	
14		90	17.20465053	
15		14.7566114	20.61552813	
16		90	13.89244399	
17		90	19.41648784	
18		90	14.86606875	
19		90	12.36931688	
20		16.8338943	10.77032961	
21		90	20.51828453	
22		18.1815264	20.1246118	
23		90	10.77032961	
24		90	14.31782106	
25		90	15.65247584	
26		21.9801816	31.01612484	
27		90	14.86606875	
28		90	17.08800749	
29		90	11.18033989	
30		14.5291923	13	
31		9.44958298	16	
32		5.93991635	21.21320344	
33		7.51610627	28.17800561	
34		28.3206857	14.86606875	
35		22.7155238	14.76482306	
36		26.4465712	13.89244399	
37		90	10.29563014	
38		90	12.64911064	
39		75.7023703	11.18033989	
40		36.3498045	16.55294536	
41		90	10.77032961	
42		7.84867287	18	
43		34.4706036	23.02172887	
44		56.2648282	13.60147051	
45		90	10.29563014	
46		90	11.70469991	
47		11.3925882	11.18033989	
48				
49				

Y Image simulation result of image L
Z Table result of image L
